# Social media posts and online search behaviour as early-warning system for MRSA outbreaks

**DOI:** 10.1186/s13756-018-0359-4

**Published:** 2018-05-30

**Authors:** Tom H. van de Belt, Pieter T. van Stockum, Lucien J. L. P. G. Engelen, Jules Lancee, Remco Schrijver, Jesús Rodríguez-Baño, Evelina Tacconelli, Katja Saris, Marleen M. H. J. van Gelder, Andreas Voss

**Affiliations:** 1 Radboud REshape Innovation Center, Radboudumc University Medical Center, Geert Grooteplein Zuid 10, 6525 GA Nijmegen, Netherlands; 2 VetEffecT, Bilthoven, The Netherlands; 3 Unidad Clínica de Enfermedades Infecciosas y Microbiología Instituto de Biomedicina de Sevilla (IBiS) /Hospital Universitario Virgen Macarena / CSIC / Departamento de Medicina, Universidad de Sevilla, Sevilla, Spain; 4 Division of Infectious Diseases, Tübingen University Hospital, DZIF Center, Tübingen, Germany; 5 Infectious Diseases, University of Verona, Verona, Italy; 6 Department of Medical Microbiology, Radboudumc, Nijmegen, The Netherlands; 7 Department for Health Evidence, Radboud Institute for Health Sciences, Radboudumc, Nijmegen, The Netherlands; 8 Department of Clinical Microbiology and Infectious Diseases, Canisius-Wilhelmina Hospital, Nijmegen, The Netherlands

**Keywords:** Methicillin-resistant *Staphylococcus aureus*, MRSA, Social media monitoring, Outbreaks, Google trends, Nowcasting

## Abstract

**Background:**

Despite many preventive measures, outbreaks with multi-drug resistant micro-organisms (MDROs) still occur. Moreover, current alert systems from healthcare organizations have shortcomings due to delayed or incomplete notifications, which may amplify the spread of MDROs by introducing infected patients into a new healthcare setting and institutions. Additional sources of information about upcoming and current outbreaks, may help to prevent further spread of MDROs.

The study objective was to evaluate whether methicillin-resistant *Staphylococcus aureus* (MRSA) outbreaks could be detected via social media posts or online search behaviour; if so, this might allow earlier detection than the official notifications by healthcare organizations.

**Methods:**

We conducted an exploratory study in which we compared information about MRSA outbreaks in the Netherlands derived from two online sources, Coosto for Social Media, and Google Trends for search behaviour, to the mandatory Dutch outbreak notification system (SO-ZI/AMR). The latter provides information on MDRO outbreaks including the date of the outbreak, micro-organism involved, the region/location, and the type of health care organization.

**Results:**

During the research period of 15 months (455 days), 49 notifications of outbreaks were recorded in SO-ZI/AMR. For Coosto, the number of unique potential outbreaks was 37 and for Google Trends 24. The use of social media and online search behaviour missed many of the hospital outbreaks that were reported to SO-ZI/AMR, but detected additional outbreaks in long-term care facilities.

**Conclusions:**

Despite several limitations, using information from social media and online search behaviour allows rapid identification of potential MRSA outbreaks, especially in healthcare settings with a low notification compliance. When combined in an automated system with real-time updates, this approach might increase early discovery and subsequent implementation of preventive measures.

## Background

The Dutch healthcare system applies strict infection control guidelines regarding multi-drug resistant micro-organisms (MDROs), including the “Search & Destroy” guideline for methicillin-resistant *Staphylococcus aureus* (MRSA), which was extended to other MDROs in 2011 [1, 2]. Despite the implementation of these guidelines, outbreaks with MDROs still occur. Reasons may be a temporary lack of compliance with existing guidelines, human error, or spread from infected patients not falling into a high-risk category that would warrant screening and isolation on admission. One of the defined high-risk-categories of the Dutch MRSA/MDRO guideline, are patients originating from a healthcare setting with an ongoing MRSA/MDRO outbreak. In the past, hospitals were supposed to inform each other about outbreaks and possible colonized or infected patients they exchange, but the report itself as well as the way of communication were non-standardized and voluntarily. As of 2012, all hospitals report their MDRO outbreaks to a central point (SO-ZI/AMR), which was initiated and established by the Dutch Society of Clinical Microbiology (NVMM) after the first carbapenem-resistant Enterobacteriaceae (CRE)-outbreak in the Netherlands [3]. SO-ZI/AMR contains a database with information about the outbreak such as date and duration of the outbreak, organization name affected location(s) and the micro-organism in question. Outbreaks that need to be reported to SO-ZI/AMR are defined as: Outbreaks which influence, or have the potential to negatively influence, access to care, such as in case of (possible) closure of a department or part of it, and/or outbreaks with continuous transmission despite (infection) control measures [4].

When reporting outbreaks became part of the professional guidelines, it became essentially mandatory. However, reporting outbreaks is currently only mandatory for hospitals, and not for nursing homes or other health care institutions. Once reported, an outbreak is, with a short delay, immediately visible for all users. The task of SO-ZI/AMR is not only to collect reports and report outbreaks to professionals, but also to monitor the development of the outbreak, and, if needed, to support the control efforts. Still, the alert messages from some hospitals seem to come late or not at all, with the risk of introducing patients infected with an MDRO into a new healthcare setting without warning and increasing the probability of spreading the outbreak. Therefore, there is a need for additional sources of information about current and potentially upcoming outbreaks, to increase outbreak preparedness.

Since an increasing number of people use social media, such as Facebook and Twitter, to share information and the Internet as source for news, social media posts and online search behaviour (e.g., via search engines) could be a valuable source of information about potential MDRO outbreaks. Interestingly, online search behaviour has already successfully been used to detect influenza outbreaks based on search entries [5], and disease outbreaks in general [6]. Moreover, social media have been used to monitor the quality of healthcare institutions on, for example, hygiene and expertise [7]. The aim of the current study was to evaluate whether Dutch MRSA outbreaks could be detected via social media posts or online search behaviour; and if so, whether these data sources might allow earlier detection than the official notification to SO-ZI/AMR by the hospital. In addition, as reporting outbreaks in nursing homes is still voluntary, we also evaluated whether screening of social media posts and/or online search behaviour would help to identify outbreaks in these healthcare institutions. In this study, we focused on MRSA.

## Methods

### Design and Setting

We conducted an exploratory study in which we compared information about MRSA outbreaks derived from social media and online search behaviour to the official Dutch reference standard. MRSA specific searches were performed for the time period between January 1st, 2015 and March 31st, 2017. As reference standard for MRSA outbreaks, SO-ZI/AMR was used [8]. It provides information on official outbreaks including the date of the outbreak and the region and type of health care facility (e.g. hospital or nursing home). The geographical scope of the study was The Netherlands; therefore, we only searched using the Dutch language.

### Social media sources and online search behaviour

To capture social media posts about MRSA (all publicly shared social media posts by individuals or organizations), we used Coosto, a social media monitoring tool [9]. This tool has proven to be a valuable source of social media information and is currently in use by the Dutch government to monitor the quality of healthcare organizations [7]. It provides the exact time and, if available, the location of the message in various social media sources, including Facebook and Twitter. Presently, its database includes posts in the Dutch language.

In addition, Google Trends was used to assess online search behaviour [10]. This tool provides insight into the search behaviour based on specific searches performed in Google Search. It provides the relative frequency of searches for different countries and regions. Google Trends has been used for early detection of influenza outbreaks [6]. Although multiple search engines are being used in The Netherlands, we limited our searches to Google Trends since Google covers over 80% of Internet searches, capturing the overall majority of Internet searches [11].

### Data extraction

We searched Coosto for publicly available social media posts about MRSA with the following search query: (“mrsa” OR “methicillin-resistant *Staphylococcus aureus*” OR “methicillin resistant *Staphylococcus aureus*” OR “meticilline-resistente *Staphylococcus aureus*” OR “meticilline resistente *Staphylococcus aureus*”). Several preliminary searches revealed that both Dutch and English names had to be included, since English terminology was sometimes used in Dutch social media posts and the combination maximized the number of hits. Furthermore, the word ‘outbreak’ was not included in the search query, since the preliminary searches showed that searching for ‘outbreak’ resulted in a large number of irrelevant hits, and that combining ‘outbreak’ with MRSA via Boolean search (“AND”) negatively affected the sensitivity of the search. Based on the results of the preliminary searches, we performed manual inspections of results with 25, 15 and 10 hits per day. In general, the lowest number of hits per day would result the highest number of potential outbreaks. However, from the above comparison, we concluded found that 10 posts per day was the minimum number of hits, or ‘critical mass’ [7] to identify a potential MRSA outbreak, Consequently, we set (≥10 hits) as criterion for a potential outbreak. For all posts on a specific day, that met this criterion, we identified whether a potential outbreak was discussed, meaning that MRSA was mentioned in relation to a Dutch healthcare institution and indicating a present or potential outbreak. The latter could consist of (but not limited to) patients, relatives or employees found or suspected with MRSA, hospital wards closed due to MRSA, any other information about a present or expected MRSA outbreak shared by the institution, its employees, government, or other any other individual (e.g., patients or relatives). Days meeting all criteria were marked as ‘representing a potential MRSA outbreak’ and all other days as ‘not representing a potential MRSA outbreak’. Dates, number of hits, institution and geographical area and outbreak (YES/NO) were subsequently stored in a research database.

Regarding the searches in Google Trends, we used similar terms, but with individual searches for each term. Google Trends presents search interest of topics on a scale from 0 to 100 per day instead of the absolute number of searches, thus every search will have at least one day with the maximum score of 100, even when absolute search numbers are low during a particular time period (e.g., a time period without any outbreaks). This system characteristic required a different way of defining days with potential outbreaks. Assuming that a Dutch MRSA outbreak occurred at least once every 3 months, we used the mean search interest of 3 months in our analyses. We extracted search interest per day, as well as geographical information (province) for days with potential outbreaks.

### Statistical analysis

All statistical analyses were done in SPSS version 22. The database with search results from Coosto and Google Trends was compared to data from the official SO-ZI/AMR database. In case of consecutive days with the same potential outbreak, we defined this as a single outbreak, both for Coosto and Google Trends. To assess the validity of Coosto to detect potential MRSA outbreaks, we calculated the overall sensitivity, specificity, positive predictive value (PPV), and negative predictive value (NPV) with 95% confidence intervals (CIs). Furthermore, we stratified the analyses by type of healthcare institution affected.

For the Google Trends data, a search score was calculated for each day:

Google Trends Score = (relative search volume - mean relative search volume) / standard deviation.

with the mean relative search volume and standard deviation based on the preceding 3 months. Using SO-ZI/AMR as the reference standard, we calculated the area under the curve (AUC) for 7 days before until 7 days after an outbreak. Furthermore, as the optimal cut-off value to detect an outbreak based on online search behaviour is unknown, we determined the Google Trends Score that maximized the sum of sensitivity and specificity. For the present study, we used a cut-off value of 2*SD to detect a potential outbreak with Google Trends. Finally, we calculated the Pearson correlation coefficient to assess the association between the number of posts related to MRSA detected with Coosto and the Google Trends Score.

## Results

During the research period of 15 months (455 days), 49 outbreaks were reported to SO-ZI/AMR (Table 1). Using Coosto, 37 potential outbreaks were detected based on social media posts, of which 1 referred to an academic hospital, 17 to a general hospital, 16 to a nursing home, and 3 to other types of institutions. Google Trends resulted in 24 potential outbreaks.

**Table 1:** Characteristics of MRSA outbreaks detected in SO-ZI/AMR and Coosto

	SO-ZI/AMR	Coosto	Google Trends
Number of days with ≥10 posts		297	
Excluded		260	
Multiple days		22	
New research		33	
Unrelated messages		59	
General info concerning MRSA		115	
Articles about hygiene and meat		31	
Total number of outbreaks	49	37	24
University hospital	8	1	
General hospital	22	17	
Nursing home	7	16	
Other	3	3	
Unknown	9	0	

**Fig. 1:**
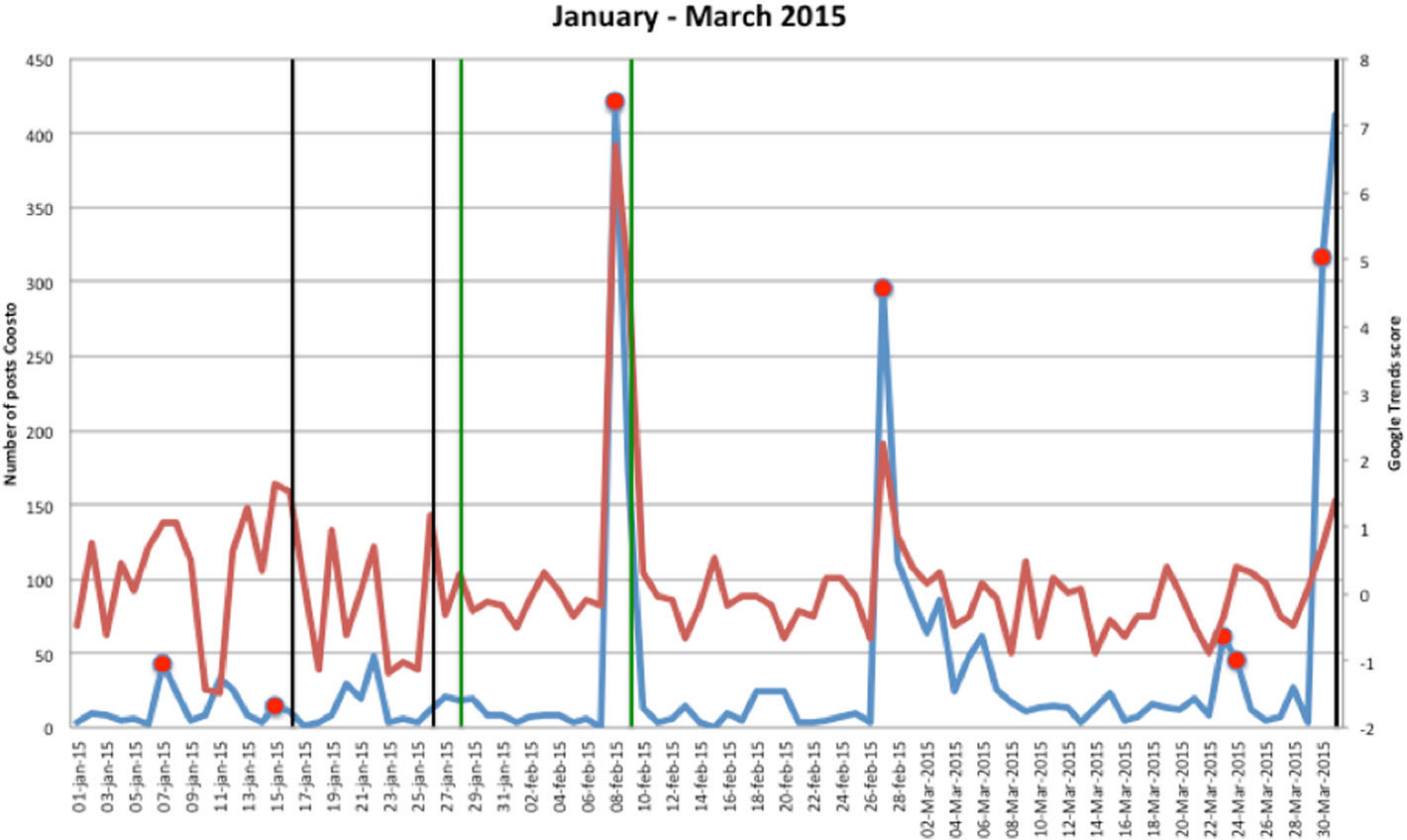
MRSA outbreaks in The Netherlands, per quarter of a year. The blue line is the number related to MRSA detected by Coosto, with a red dot indicating days with ≥10 posts related to an outbreak, the red line the Google Trends score. The vertical bars represent outbreaks reported to SO-ZI/AMR: blue for teaching hospitals, black for regular hospitals, green for nursing homes, yellow for other locations, and red for unknown locations

**Fig. 2:**
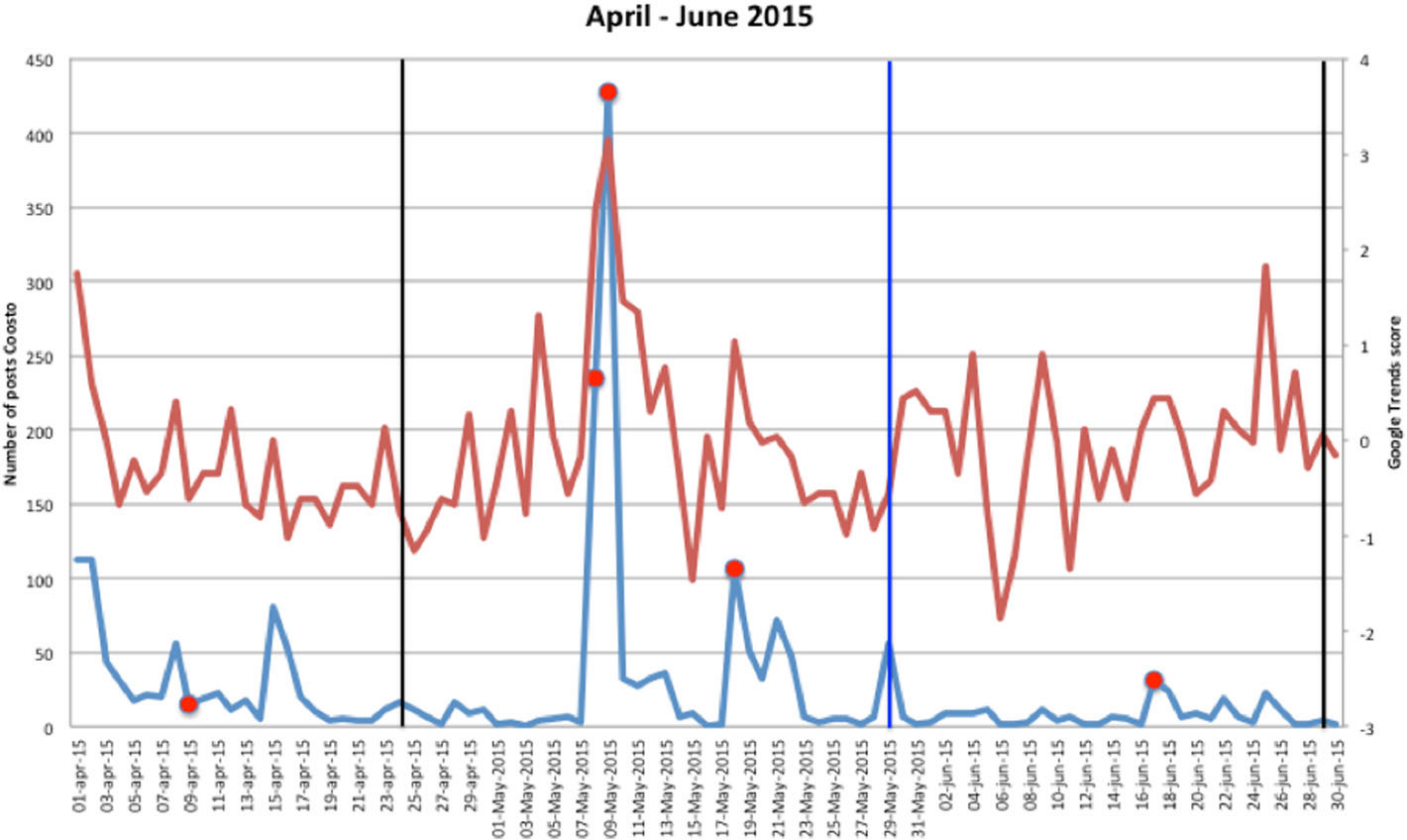
MRSA outbreaks in The Netherlands, per quarter of a year. The blue line is the number related to MRSA detected by Coosto, with a red dot indicating days with ≥10 posts related to an outbreak, the red line the Google Trends score. The vertical bars represent outbreaks reported to SO-ZI/AMR: blue for teaching hospitals, black for regular hospitals, green for nursing homes, yellow for other locations, and red for unknown locations

**Fig. 3:**
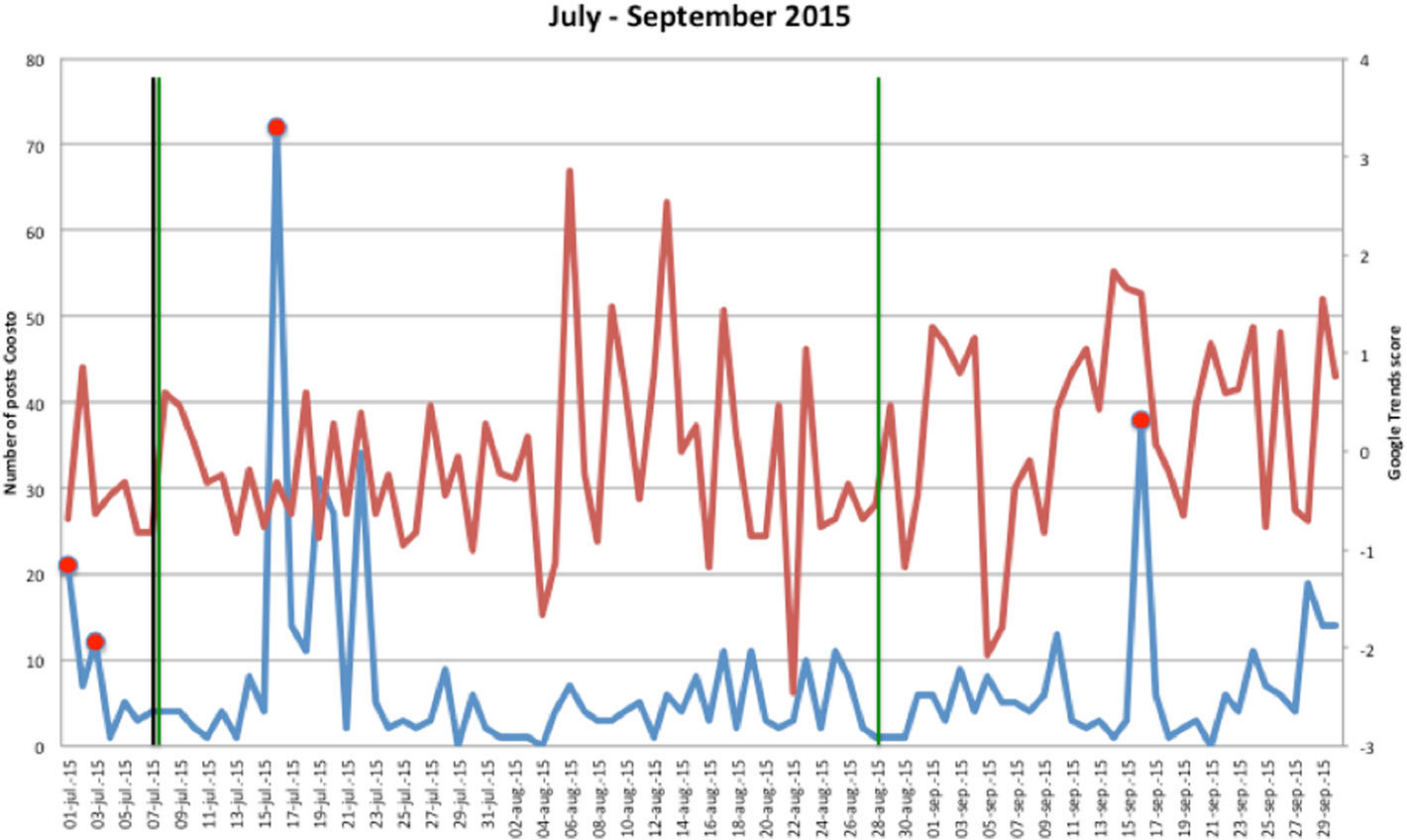
MRSA outbreaks in The Netherlands, per quarter of a year. The blue line is the number related to MRSA detected by Coosto, with a red dot indicating days with ≥10 posts related to an outbreak, the red line the Google Trends score. The vertical bars represent outbreaks reported to SO-ZI/AMR: blue for teaching hospitals, black for regular hospitals, green for nursing homes, yellow for other locations, and red for unknown locations

**Fig. 4:**
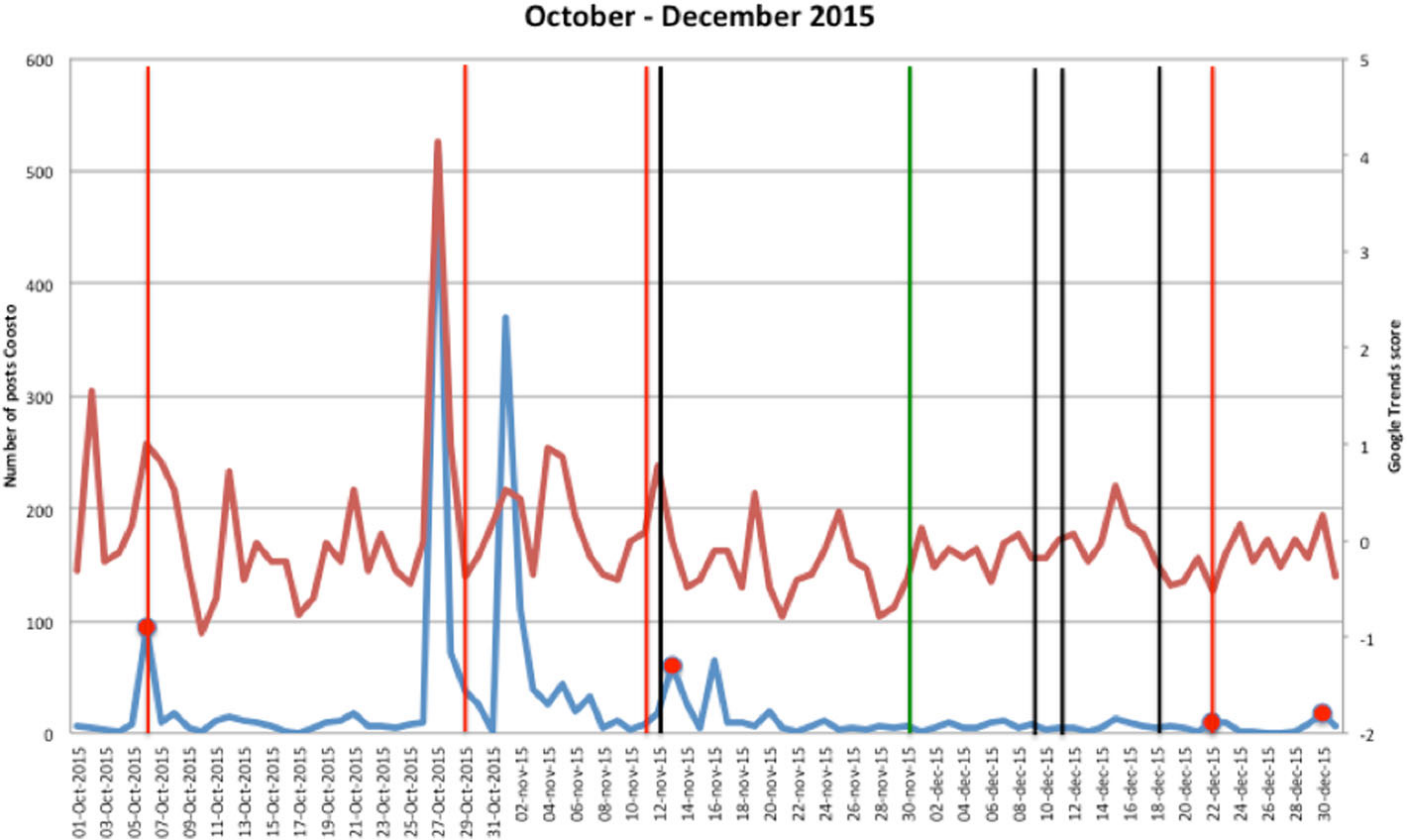
MRSA outbreaks in The Netherlands, per quarter of a year. The blue line is the number related to MRSA detected by Coosto, with a red dot indicating days with ≥10 posts related to an outbreak, the red line the Google Trends score. The vertical bars represent outbreaks reported to SO-ZI/AMR: blue for teaching hospitals, black for regular hospitals, green for nursing homes, yellow for other locations, and red for unknown locations

**Fig. 5:**
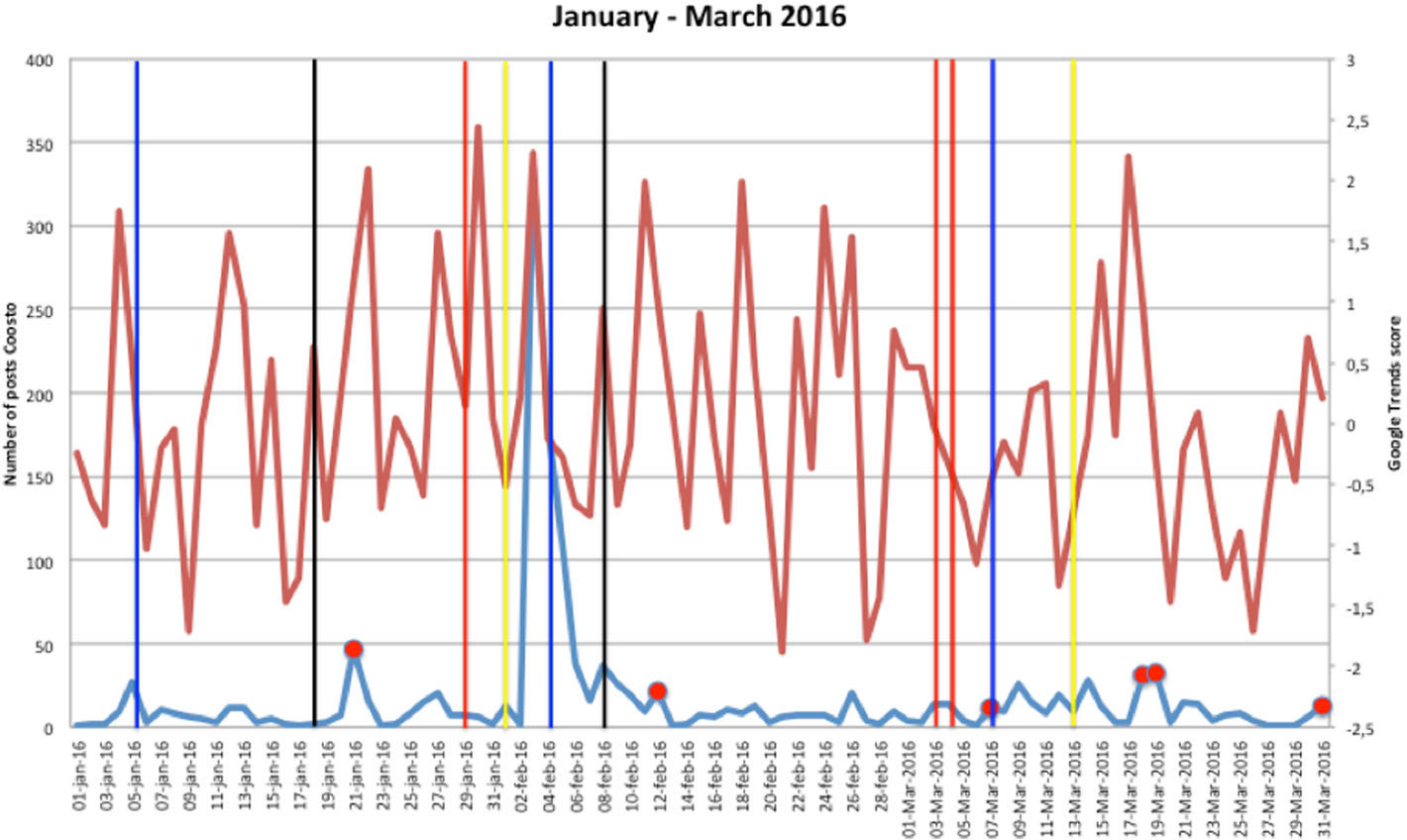
MRSA outbreaks in The Netherlands, per quarter of a year. The blue line is the number related to MRSA detected by Coosto, with a red dot indicating days with ≥10 posts related to an outbreak, the red line the Google Trends score. The vertical bars represent outbreaks reported to SO-ZI/AMR: blue for teaching hospitals, black for regular hospitals, green for nursing homes, yellow for other locations, and red for unknown locations

**Fig. 6:**
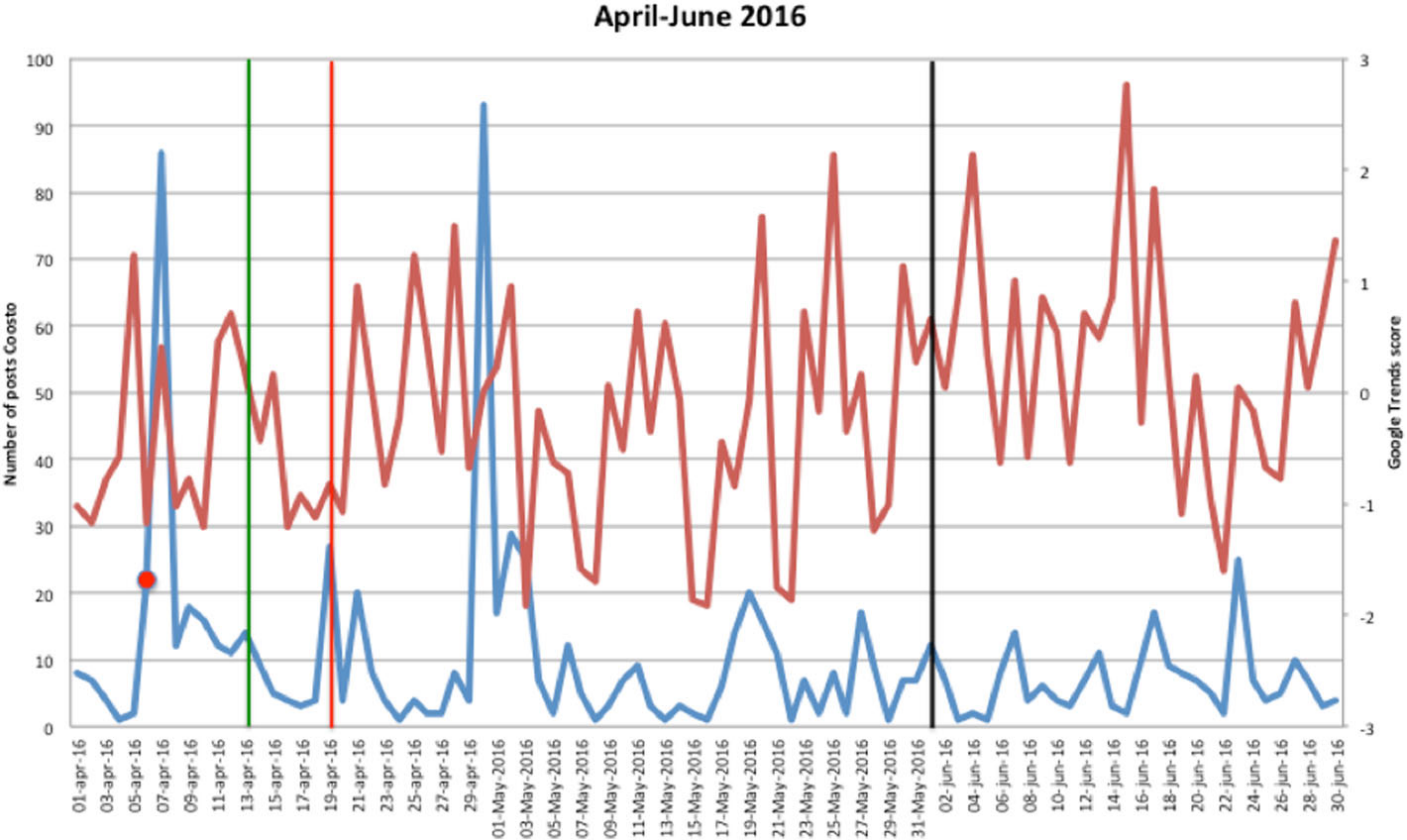
MRSA outbreaks in The Netherlands, per quarter of a year. The blue line is the number related to MRSA detected by Coosto, with a red dot indicating days with ≥10 posts related to an outbreak, the red line the Google Trends score. The vertical bars represent outbreaks reported to SO-ZI/AMR: blue for teaching hospitals, black for regular hospitals, green for nursing homes, yellow for other locations, and red for unknown locations

**Fig. 7:**
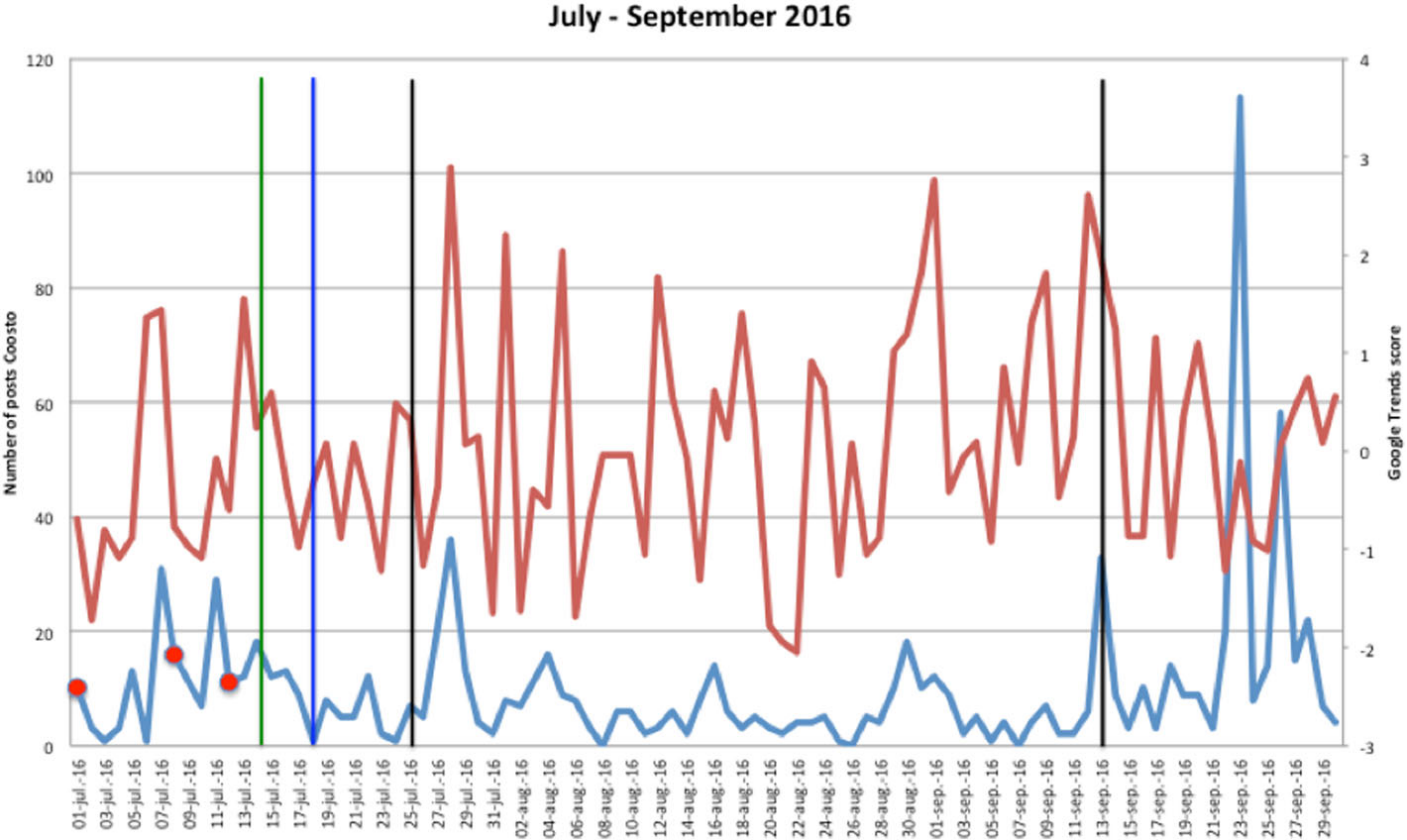
MRSA outbreaks in The Netherlands, per quarter of a year. The blue line is the number related to MRSA detected by Coosto, with a red dot indicating days with ≥10 posts related to an outbreak, the red line the Google Trends score. The vertical bars represent outbreaks reported to SO-ZI/AMR: blue for teaching hospitals, black for regular hospitals, green for nursing homes, yellow for other locations, and red for unknown locations

**Fig. 8:**
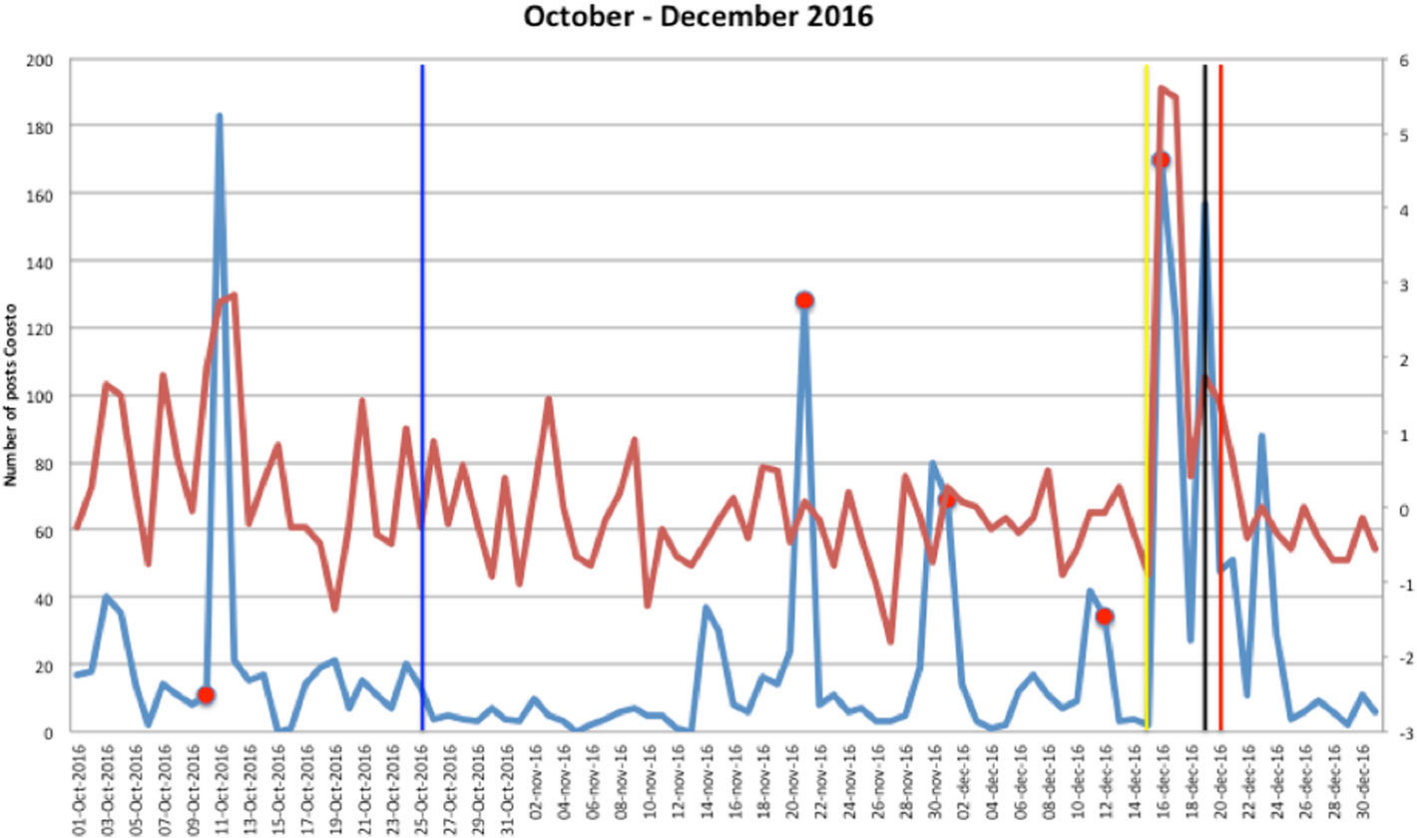
MRSA outbreaks in The Netherlands, per quarter of a year. The blue line is the number related to MRSA detected by Coosto, with a red dot indicating days with ≥10 posts related to an outbreak, the red line the Google Trends score. The vertical bars represent outbreaks reported to SO-ZI/AMR: blue for teaching hospitals, black for regular hospitals, green for nursing homes, yellow for other locations, and red for unknown locations

**Fig. 9:**
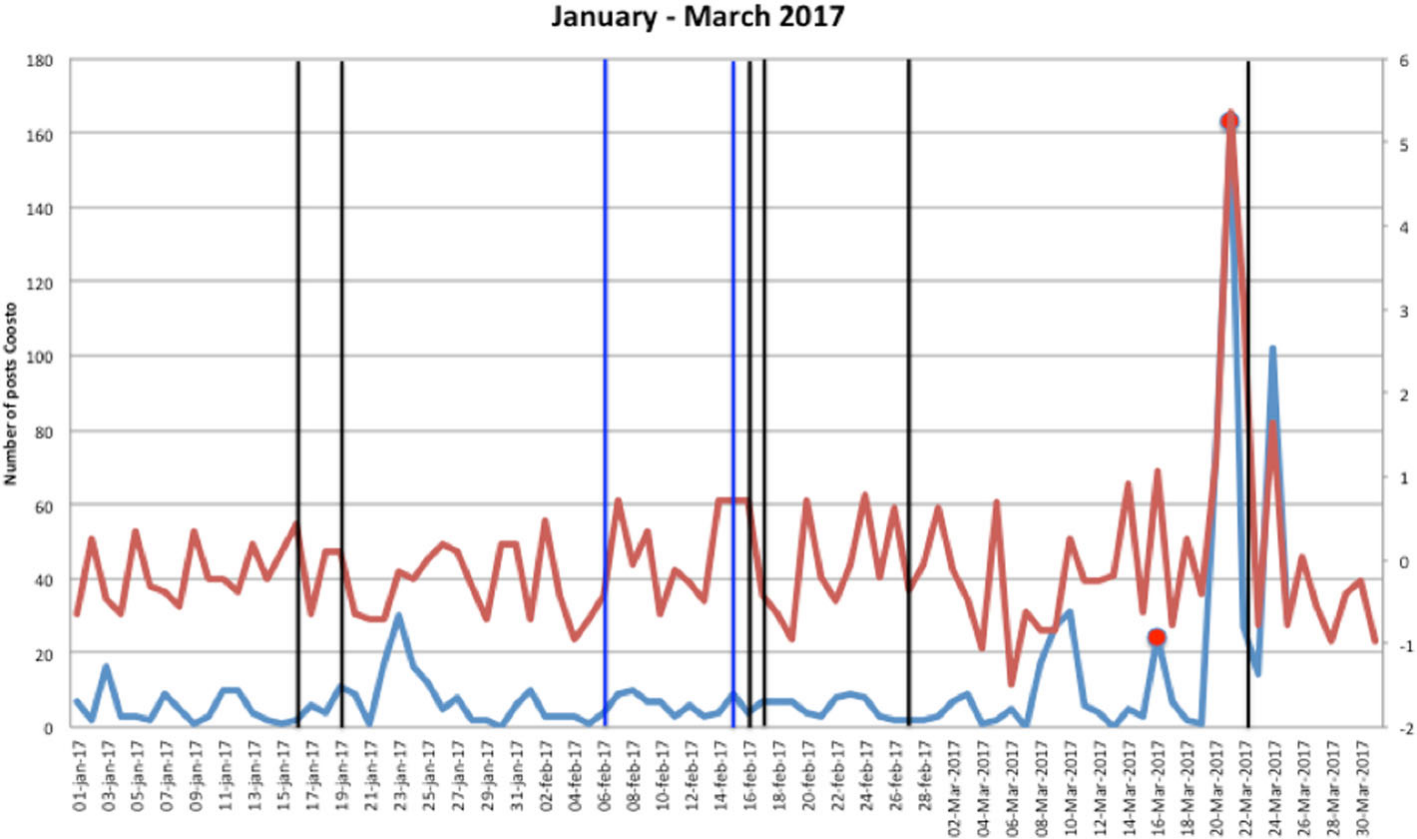
MRSA outbreaks in The Netherlands, per quarter of a year. The blue line is the number related to MRSA detected by Coosto, with a red dot indicating days with ≥10 posts related to an outbreak, the red line the Google Trends score. The vertical bars represent outbreaks reported to SO-ZI/AMR: blue for teaching hospitals, black for regular hospitals, green for nursing homes, yellow for other locations, and red for unknown locations

Figures 1, 2, 3, 4, 5, 6, 7, 8, and 9 show the information on MRSA outbreaks originating from the three data sources in each quarter of a year. In only 4 outbreaks did all three sources show a (potential) outbreak, with in general the online sources detecting the outbreak 1–2 days before the official notification date in SO-ZI/AMR. In 48 cases, Coosto and/or Google Trends indicated a (potential) outbreak without notification in SO-ZI/AMR, whereas in 41 cases, MRSA outbreaks were notified to SO-ZI/AMR that went unnoticed by the online data sources. In 4 cases, a (potential) outbreak was detected by both SO-ZI-AMR and Coosto, and not by Google Trends. Correlation between the number of posts related to MRSA detected with Coosto and the Google Trends Score was 0.42, *p* < 0.001).

Validity comparisons for Coosto-detected MRSA outbreaks showed an overall sensitivity of 0.20 (95% CI 0.10–0.34) and an overall specificity of 0.96 (95% CI 0.95–0.98), whereas the PPV and NPV were 0.27 (95% CI 0.16–0.42) and 0.95 (95% CI 0.92–0.96), respectively (Table 2). After stratification for type of healthcare institution, sensitivity ranged between 0 for other and unknown locations to 0.23 (95% CI 0.08–0.45) for general hospitals. Specificity was ≥0.98 for all types of institutions.

**Table 2:** Validity comparisons for Coosto-detected MRSA outbreaks with SO-ZI/AMR as the reference standard

MRSA outbreaks	Number of days					Validity							
	TP	FP	FN	TN		Se	(95% CI)	Sp	(95% CI)	PPV	(95% CI)	NPV	(95% CI)
All	10	27	39	722		20	(10–34)	96	(95–98)	27	(16–42)	95	(92–96)
Any hospital	5	13	25	755		17	(6–35)	98	(97–99)	28	(13–50)	97	(96–97)
University hospital	0	1	8	791		0		100	(99–100)	0		99	(99–99)
General hospital	5	12	17	764		23	(8–45)	98	(97–99)	29	(14–52)	98	(97–98)
Any hospital and unknown location	6	12	33	747		15	(6–31)	98	(97–99)	33	(17–56)	96	(95–96)
University hospital and unknown location	0	1	17	783		0		100	(99–100)	0		98	(98–98)
General hospital and unknown location	6	11	25	756		19	(7–37)	99	(97–99)	35	(18–58)	97	(96–97)
Nursing home	1	15	6	778		13	(0–53)	98	(97–99)	6	(1–31)	99	(99–99)
Nursing home and unknown location	2	14	14	770		13	(2–38)	98	(97–99)	13	(3–37)	98	(98–99)
Other location	0	3	3	795		0		100	(99–100)	0		100	(100–100)
Other location and unknown location	0	3	12	786		0		100	(99–100)	0		99	(98–99)

*CI* confidence interval, *FN* false negative, *FP* false positive, *NPV* negative predictive value, *PPV* positive predictive value, *Se* sensitivity, *Sp* specificity, *TN* true negative, *TP* true positive

The validity comparisons for Google Trends to detect MRSA outbreaks compared with SO-ZI/AMR as the reference standard are shown in Table 3. On the exact date the outbreak was notified to SO-ZI/AMR, the AUC was 0.59 (95% CI 0.51–0.67) for any MRSA outbreak and 0.63 (95% CI 0.54–0.73) for MRSA outbreaks in hospitals. With the optimal cut-off for the Google Trends Score, sensitivity was higher for any outbreak compared with hospital outbreaks only (0.90 vs. 0.43), whereas specificity was higher for hospital outbreaks (0.28 vs. 0.79). The AUC based on the Google Trends Score 1 day before the official notification was similar to the AUC on the day of notification. On the other days relative to the notification of the outbreaks, the AUC was decreased.

**Table 3:** Validity comparisons for Google Trends to detect MRSA outbreaks with SO-ZI/AMR as the reference standard. Day 0 indicates the day the outbreak was reported to SO-ZI/AMR

Day	Any MRSA outbreak					MRSA outbreak in hospital				
	AUC	(95% CI)	Optimal cutoff^a^	Se	Sp	AUC	(95% CI)	Optimal cutoff^a^	Se	Sp
−7	0.61	(0.54–0.69)	−0.2018	75	47	0.59	(0.51–0.67)	− 0.6507	100	23
−6	0.51	(0.44–0.59)	− 0.1208	63	51	0.51	(0.42–0.59)	−0.6965	93	21
−5	0.46	(0.38–0.55)	−0.4318	71	33	0.48	(0.39–0.58)	−0.8801	97	13
−4	0.50	(0.42–0.58)	−0.5524	81	28	0.53	(0.44–0.62)	−0.5524	87	28
−3	0.46	(0.38–0.54)	−0.5377	81	29	0.48	(0.39–0.58)	−0.5377	87	29
−2	0.52	(0.44–0.61)	0.1445	42	65	0.47	(0.37–0.57)	−0.6828	83	21
−1	0.59	(0.50–0.67)	0.0425	60	61	0.59	(0.47–0.71)	0.0672	67	62
0	0.59	(0.51–0.67)	−0.5524	90	28	0.63	(0.54–0.73)	0.3316	43	79
+ 1	0.54	(0.46–0.63)	−0.1769	63	48	0.49	(0.39–0.59)	−1.1493	100	7
+ 2	0.49	(0.41–0.57)	− 1.1833	100	6	0.46	(0.36–0.55)	−0.9484	97	11
+3	0.50	(0.43–0.58)	−0.5149	79	29	0.56	(0.46–0.66)	−0.1118	63	51
+ 4	0.49	(0.41–0.57)	−0.6828	90	21	0.49	(0.40–0.59)	−0.6828	93	21
+ 5	0.44	(0.37–0.51)	0.1674	15	65	0.39	(0.31–0.47)	−1.3370	100	4
+ 6	0.49	(0.42–0.57)	− 1.0128	98	9	0.47	(0.38–0.57)	−1.0128	97	9
+ 7	0.53	(0.45–0.60)	−0.4993	85	30	0.50	(0.40–0.61)	− 0.5745	83	27

*AUC* area under the curve, *CI* confidence interval, *Se* sensitivity, *Sp* specificity

^a^Maximizes sensitivity+specificity. Value represents k

## Discussion

### Principal findings

In this study, we compared information about potential MRSA outbreaks retrieved from social media posts and online search behaviour in The Netherlands to the national notification reference standard. We found that simple online (social media) searches do provide additional information about potential MRSA outbreaks in The Netherlands compared to the reference standard. These promising findings suggest that supervisory bodies such as SO-ZI/AMR may enrich their palette of data sources with more dynamic information from social media and other online sources such as search engine data. However, the validity of the online sources Coosto and Google trends needs further investigation. Some things need to be discussed.

The sensitivity of the social media monitoring tool Coosto to detect MRSA outbreaks was low and therefore a substantial number of true outbreaks will be missed when relying on this data source. However, its specificity was high, indicating a relatively small number of false positive outbreaks detected by Coosto. Interestingly, the opposite was observed for Google Trends, with a higher sensitivity and lower specificity, indicating that Google Trends detects more potential MRSA outbreaks but that many of these represent false positive signals. This difference between the two online data sources may be explained by their nature: social media posts provide more detailed information on MRSA outbreaks than online searches, but the patients and healthcare workers involved in MRSA outbreaks may be more reluctant to post a message about the outbreak on social media than to search for information online. In addition, it is impossible to distinguish between online searches for actual outbreaks and searches for random issues related to MRSA using Google Trends.

To the best of our knowledge, this was the first study using online search engines for social media posts and internet search behaviour on MRSA outbreak detection. A study by Lui et al. used search terms from the social media platform Baidu to identify Noro virus epidemics [12]. They concluded that several limitations exist to using Internet to monitor epidemics but that it still might have value as additional tool, particularly when other monitoring systems are lacking. Also, the importance of social media as an early warning system in addition to traditional slow reporting mechanisms has been emphasized [13]. In general, this might be the case for all outbreaks as notification is only done after firm confirmation of the outbreak. Consequently, the notification date in SO-ZI/AMR is “delayed” by several days.

Additional potential outbreaks were found via social media, which were not in the SO-ZI/AMR database. Most of these outbreaks occurred in nursing homes for which notification is not mandatory, but on occasion even hospitals were shown as non-compliant in reporting outbreaks. The fact that more dynamic data sources could have value compared to traditional slow reporting mechanisms has also been recognized in public health, where social media are used as an early warning system for disease outbreaks [14].

### Strengths and limitations

The main strength of this study is the use of dynamic online content from social media and search engine behaviour in combination with an official reference (SO-ZI/AMR). Using predefined selection criteria, this allowed us to efficiently study the value of social media and online search behaviour via both Coosto and Google Trends. Coosto on occasion is limited by the fact that it may not always be possible to determine whether a potential outbreak is actually a true outbreak. Google Trends has even more difficulty in this determination, for using this data source, it is hard to link potential outbreaks to specific organizations, since it does not provide specific names or locations. A refinement of search mechanism of the freely available default Google Trends software might allow an increase of its sensitivity and specificity.

The extent of information patients and caregivers get when confronted with an outbreak may influence their search behaviour. If the information is complete and offered right away, as it is customary in many Dutch hospitals, it may become more difficult to detect the outbreak using social media and search engine behaviour.

## Conclusions

Despite several limitations including limited validity, using information from social media and online search behaviour results to detect MRSA outbreaks could be an additional source of information for supervising bodies, particularly when combined in an automated system with real-time updates.
